# Immuno-fluorescent Labeling of Microtubules and Centrosomal Proteins in *Ex Vivo* Intestinal Tissue and 3D *In Vitro* Intestinal Organoids

**DOI:** 10.3791/56662

**Published:** 2017-12-13

**Authors:** Deborah A. Goldspink, Zoe J. Matthews, Elizabeth K. Lund, Tom Wileman, Mette M. Mogensen

**Affiliations:** ^1^School of Biological Sciences, University of East Anglia; ^2^Norwich Medical School, University of East Anglia

**Keywords:** Developmental Biology, Issue 130, Organoid, intestine, cytoskeleton, microtubules, EB1, CLIP-170, ninein, centrosome, Lgr5, epithelia

## Abstract

The advent of 3D *in vitro* organoids that mimic the *in vivo* tissue architecture and morphogenesis has greatly advanced the ability to study key biological questions in cell and developmental biology. In addition, organoids together with recent technical advances in gene editing and viral gene delivery promises to advance medical research and development of new drugs for treatment of diseases. Organoids grown *in vitro* in basement matrix provide powerful model systems for studying the behavior and function of various proteins and are well suited for live-imaging of fluorescent-tagged proteins. However, establishing the expression and localization of the endogenous proteins in *ex vivo* tissue and in *in vitro* organoids is important to verify the behavior of the tagged proteins. To this end we have developed and modified tissue isolation, fixation, and immuno-labeling protocols for localization of microtubules, centrosomal, and associated proteins in *ex vivo* intestinal tissue and in *in vitro* intestinal organoids. The aim was for the fixative to preserve the 3D architecture of the organoids/tissue while also preserving antibody antigenicity and enabling good penetration and clearance of fixative and antibodies. Exposure to cold depolymerizes all but stable microtubules and this was a key factor when modifying the various protocols. We found that increasing the ethylenediaminetetraacetic acid (EDTA) concentration from 3 mM to 30 mM gave efficient detachment of villi and crypts in the small intestine while 3 mM EDTA was sufficient for colonic crypts. The developed formaldehyde/methanol fixation protocol gave very good structural preservation while also preserving antigenicity for effective labeling of microtubules, actin, and the end-binding (EB) proteins. It also worked for the centrosomal protein ninein although the methanol protocol worked more consistently. We further established that fixation and immuno-labeling of microtubules and associated proteins could be achieved with organoids isolated from or remaining within the basement matrix.

**Figure:**
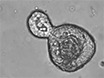


## Introduction

Formation of epithelia with apico-basal polarity is a fundamental process in development and involves a dramatic reorganization of the microtubules and centrosomal proteins. A radial microtubule array emanating from a centrally located centrosomal microtubule organizing center (MTOC) is prominent in many animal cells and this is well suited for relatively flat cells. In contrast, columnar epithelial cells, such as those of the intestine, assemble non-radial transcellular microtubule arrays that better support the shape and specialized functions of these cells. This dramatic reorganization of the microtubules is achieved by the centrosome moving to the apex and apical non-centrosomal MTOCs (n-MTOCs) forming, which becomes responsible for anchorage of the transcellular microtubules^1^,^2^,^3^,^4^,^5^.

Much of our knowledge of epithelial differentiation and the associated microtubule reorganization has come from investigations of 2D *in vitro* cell layers that do not display the *in vivo* tissue architecture. Development of 3D *in vitro* organoid cultures, pioneered by Clevers and co-workers^6^, represents a major technological advancement as they mimic *in vivo* architecture and development. A hierarchy of epithelial differentiation is evident in the intestine; stem cells at the bottom of crypts give rise to immature transit amplifying cells that proliferate and gradually differentiate as they migrate up the crypt onto the small intestinal villus or colonic surface, where they become fully differentiated prior to being shed into the lumen^7^. Importantly, this is replicated in intestinal organoids where cells from the stem cell niche proliferate forming cysts that subsequently generate crypt-like buds with stem cells at the bottom and differentiation gradually progressing towards the cyst region, which becomes villus-like^8^. The intestinal organoid therefore represents a powerful model to study not only microtubule and centrosomal reorganization during epithelial differentiation but numerous other proteins, as well as providing an ideal platform for screening of drugs and food compounds of potential therapeutic benefits^9^,^10^.

Organoids are well suited for live-imaging of fluorescent-tagged proteins and both knock-in and knock-out organoids can be generated using CRISPR/Cas9 gene editing^11^,^12^. However, establishing the expression and localization of the endogenous proteins to be studied is important, especially to verify the behavior of the tagged proteins. Immuno-labeling 3D organoids grown in basement matrix or *ex vivo* isolated tissue is more complex than cells grown in culture dishes in 2D. The fixation protocol needs to preserve the delicate 3D architecture of organoids while still preserving antibody antigenicity (*i.e.*, the epitopes for binding antibodies). For example, 4% paraformaldehyde (PFA) is commonly used as a fixative but while it is a relatively rapid acting fixative and gives good morphological preservation, in our experience it frequently results in loss of antigenicity and is not suitable for many centrosomal antibodies. The ability of the fixative and antibodies to penetrate 3D structures and tissue should also be considered. To this end, we have modified and developed protocols for tissue isolation and indirect immuno-labeling of 3D *in vitro* organoids and *ex vivo* isolated intestinal tissue. We describe how to isolate small intestinal crypts and villi and colonic tissue, and include a protocol for isolation of 3D organoids as an alternative to fixing and immuno-labeling within the basement matrix. We present three alternative fixation protocols for immuno-labeling of microtubules and centrosomal proteins, such as ninein, and microtubule plus-end tracking proteins (+TIPs), such as the EB proteins and CLIP-170 (see also references^8^,^13^). We also discuss the pros and cons associated with each protocol.

## Protocol

All methods described here were performed according to the University of East Anglia's institutional license guidelines.

### 1. Isolation of Intestinal Tissue

1. **Isolation of colonic crypts for immuno-labeling (see Figure 1, schematic)**
  1. Euthanize the mouse (using CO_2_ asphyxiation) and remove the colon (beginning at the caecum and extracting caudally) with dissecting scissors and tweezers^14^.
  2. Flush the content of the colon with phosphate buffered saline (PBS) using a glass pipette with rubber bulb. PBS: sodium chloride (8.0 g/L), potassium chloride (0.2 g/L), disodium hydrogen phosphate (1.15 g/L), and potassium dihydrogen phosphate (0.2 g/L), at pH 7.3.
  3. Using a metal rod (2.4 mm diameter) or the end of a glass pipette, hold one end of the colonic tube while gently sliding the tissue over the rod/pipette thus everting the colonic tissue so that the epithelium is now on the outside. NOTE: If this is not possible then open the colon with scissors and slice into 5 mm pieces.
  4. Transfer the everted colon or pieces to a 50 mL conical tube containing 40 mL of PBS.
  5. Invert the tube several times to further remove intestinal contents, mucus, *etc.*
  6. Transfer the colon/pieces to 40 mL of 3 mM EDTA in PBS and incubate at room temperature (RT) for 15 min. NOTE: Dilute 0.5 M EDTA pH 8.0 stock with PBS.
  7. Shake to remove the mucus and transfer the colon/pieces to a 50 mL conical tube containing fresh 40 mL of 3 mM EDTA in PBS. Incubate for 35 min at RT.
  8. Transfer the colon/pieces to 10 mL of PBS in a 50 mL conical tube and shake vigorously for 30 s to release crypts (crypt fraction).
  9. Remove the colon/pieces from the tube and place back into 3 mM EDTA in case further isolation is required. Centrifuge the remaining crypt fraction at 300 x g for 5 min.
  10. Remove 5 mL of the supernatant and resuspend the crypt pellet in the remaining 5 mL volume.
  11. Observe under a stereomicroscope with zoom (50 - 100X magnification) to check for presence of colonic crypts. NOTE: If there are no crypts present then incubate the colon/pieces in 3 mM EDTA in PBS for another 30 min and then repeat steps 1.1.8 - 1.1.11.
  12. Centrifuge the crypt fraction at 300 x g for 5 min.
  13. Remove all supernatant to obtain a crypt pellet and proceed immediately to fixation (Section 3).
2. **Isolation of small intestinal crypts and villi for immuno-labeling (Figure 2)**
  1. Euthanize mouse (using CO_2_ asphyxiation) and remove the small intestine (proximal duodenum to terminal ileum) with dissecting scissors and tweezers^14^. NOTE: To view different sections of the small intestine then at this stage separate and treat separately. See Figure 1 and **Table 1** for a flow diagram and timings.
  2. Flush the contents of the small intestine with PBS using a glass pipette with rubber bulb.
  3. Cut open the small intestine with dissecting scissors and place in 15 mL PBS in a Petri dish.
  4. Gently wash the intestine in PBS to remove luminal content while not damaging the mucosal surface. Transfer the tissue to a fresh Petri dish and repeat PBS wash.
  5. Cut the intestine into 3-5 mm pieces and transfer to a 100 mm Petri dish containing 15 mL of 30 mM EDTA in PBS and incubate for 5 min at RT. Alternatively incubate in 3 mM EDTA in PBS for 30 min.
  6. Fraction 1 (Villi isolation): Transfer the intestinal pieces into 10 mL of PBS in a 50 mL conical tube, shake vigorously for 10 s, and pour into a 100 mm Petri dish. Check the fraction for isolated villi under a stereomicroscope. At this stage, mostly villi should be present but this can vary between isolations. NOTE: To prevent isolated villi/crypts sticking to the plastic, Petri dishes and collection tubes should be pre-coated with Fetal Bovine Serum (FBS). Pour FBS into dishes and/or tubes and immediately remove it. See **Table 1** for timing guides for each fraction.
  7. Transfer the intestinal pieces back into 30 mM EDTA in PBS and incubate for 5 min. During this incubation, collect Fraction 1 in a 15 mL conical tube (precoated with FBS) and centrifuge at 300 x g for 5 min.
  8. Fraction 2: Transfer intestinal pieces to a 50 mL conical tube containing 10 mL of PBS, shake vigorously for 20 s, and then pour into a 100 mm Petri dish. Check the fraction under the microscope. Typically, at this stage a mixed culture of villi and crypts are present (Figure 2A).
  9. Transfer intestinal pieces back into 30 mM EDTA in PBS and incubate for a further 5 min. During this incubation, pellet Fraction 2 in a 15 mL conical tube (precoated with FBS) at 300 x g for 5 min. Remove supernatant from Fractions 1 and 2 without disturbing the pellet and proceed immediately to fixation (step 3).
  10. Fraction 3 (Crypt isolation): Transfer the intestinal pieces into 10 mL of PBS in 50 mL tube, shake for 20 s, and pour into a 100 mm Petri dish. Check fraction under the microscope. At this stage, the fraction will contain predominantly crypts (Figure 2B).
  11. Fraction 4: Transfer the intestinal pieces into 10 mL of PBS, shake for 20 s, and pour into a 100 mm Petri dish. Check fraction under the microscope. At this stage only crypts should be present.
  12. Fraction 5: Transfer the intestinal pieces into 10 mL of PBS, shake for 20 s, and pour into a 100 mm Petri dish. Check fraction under the microscope. Usually, at this stage, very few crypts are extracted. If more than a few crypts are extracted, then continue with a 6^th^ fraction. NOTE: If a pure crypt population is desired (for example, for organoid generation), then use a 70 µm cell strainer to remove any intact villi from the crypt-enriched fraction.
  13. Collect Fractions 3 - 5 in separate 15 mL conical tubes and centrifuge at 300 x g for 5 min.
  14. Check the pellets of Fractions 3 - 5 and remove the supernatants without disturbing the pellets, and proceed immediately to fixation (Section 3).

### 2. Isolation of Intestinal Organoids from Basement Matrix Domes in 24-well Plates

NOTE: The formation of organoids within basement matrix domes has been described elsewhere^12^.

1. Coat wells with basement matrix dome according to the manufacturer's instructions.
2. Wash wells containing basement matrix domes with 500 µL of PBS.
3. Add cold 250 µL of cell recovery solution (4 °C) to each well (see **Table of Materials**). NOTE: The cold cell recovery solution will depolymerize all but stable microtubules.
4. Scrape the basement matrix domes using a P1000 micropipette and carefully pipette up and down throughout the well to breakup and remove the basement matrix from plastic.
5. Collect the supernatant in 1.5 mL low binding microcentrifuge tubes.
6. Invert the 1.5 mL low binding tube several times and check under the microscope whether the organoids have been isolated and are free moving and not in clumps. Hold the tube under a stereomicroscope and view under low (50X) magnification.
7. Pellet the organoids by centrifugation at 1,000 x g for 5 min at RT.
8. Remove the recovery reagent and proceed immediately to fixation (step 3).

### 3. Fixation of Isolated Intestinal Tissue and Organoids

1. **Methanol fixation**
  1. Resuspend the isolated intestinal crypt/villus fraction in 10 mL and organoids in 1 mL of methanol at -20 °C.
  2. Incubate the crypts/villi/organoids at -20 °C in a freezer for 15 min and invert the tube(s) every 5 min.
  3. Pellet the crypts/villi/organoids by centrifugation at 1,000 x g for 5 min.
  4. Remove the methanol and add washing solution (1 mL for organoids and 10 mL for isolated crypt/villus fractions). The washing solution may either consist of PBS with 1% serum or PBS with 0.1% detergent and 1% serum. NOTE: Use serum from the same species as that of the secondary antibodies. For example, if the secondary antibodies were generated in goat then use goat serum.
  5. Immediately, centrifuge the crypts/villi/organoids at 1,000 x g for 5 min to pellet.
  6. Remove the washing solution and resuspend the crypts/villi/organoids in fresh washing solution.
  7. Place on a tube rotator with the speed set to 20 rpm. Wash the cells for a total of 1 h, pelleting the crypts/villi/organoids by centrifugation (1,000 x g for 5 min) every 15 min and replacing the wash solution. NOTE: If a tube rotator is not available then invert the tubes manually every 5 min to resuspend the isolated cultures.
2. **Formaldehyde/methanol fixation** NOTE: Handle the fixatives and formaldehyde in a fume hood.
  1. Resuspend the isolated crypts/villi in 10 mL or organoids in 1 mL of -20 °C fixative solution (9.2 mL of methanol with 800 µL of formaldehyde solution).
  2. Fix the crypts/villi/organoids at -20 °C in a freezer for 15 min and invert the tube every 5 min.
  3. Pellet the crypts/villi/organoids by centrifugation at 1,000 x g for 5 min.
  4. Remove the formaldehyde/methanol and add washing solution (1 mL for organoids or 10 mL for isolated crypts/villus fractions). The washing solution may either consist of PBS with 1% goat serum or PBS with 0.1% detergent and 1% serum.
  5. Centrifuge at 1,000 x g for 5 min to pellet the crypts/villi/organoids.
  6. Remove the washing solution and resuspend in fresh washing solution.
  7. Place on a tube rotator with the speed set to 20 rpm. Wash the cells for a total of 1 h, pelleting the crypts/villi/organoids by centrifugation (1,000 x g for 5 min) every 15 min and replacing the wash solution.
3. **PFA fixation** CAUTION: PFA powder and solutions should be handled in a fume hood. NOTE: In most cases 4% PFA in PBS is used but depending on preservation of antibody antigenicity, lower concentrations may be used.
  1. Resuspend the isolated pelleted crypts/villi in 10 mL 4% PFA and incubate at RT for 1 h, and the isolated pelleted organoids in 1 mL 4% PFA and incubate for 30 min. In both cases invert the tube(s) every 10 min.
  2. Pellet the crypts/villi/organoids by centrifugation at 1,000 x g for 5 min.
  3. Remove the PFA and add washing solution (1 mL for organoids and 10 mL for isolated crypts/villi). The washing solution consists of PBS with 0.1% detergent and 1% serum.
  4. Centrifuge at 1,000 x g for 5 min to pellet crypts/villi/organoids.
  5. Remove the washing solution and resuspend in fresh washing solution.
  6. Place on a tube rotator with speed set to 20 rpm. Wash the cells for a total of 1 h, pelleting the crypts/villi/organoids by centrifugation (1,000 x g for 5 min) every 15 min and replacing the wash solution. NOTE: If a tube rotator is not available then invert tubes manually every 5 min to resuspend the isolated cultures.
  7. Antigen Retrieval (optional step recommended for PFA fixation)
    1. Pellet the crypts/villi/organoids by centrifugation at 1,000 x g for 5 min and remove the supernatant.
    2. Add 1 - 10 mL of 10 mM sodium citrate pH 6.0 (pre-warmed to 80 °C) and incubate at 80 °C for 20 min.
    3. Pellet the crypts/villi/organoids by centrifugation at 1,000 x g for 5 min and remove the supernatant.
    4. Add 1 - 10 mL of fresh 10 mM sodium citrate pH 6.0 (pre-warmed to 80 °C) and incubate at RT for 20 min.
    5. Pellet the crypts/villi/organoids by centrifugation at 1,000 x g for 5 min and remove the supernatant.
    6. Wash in 1 - 10 mL PBS with 1% serum and repeat a further 3 times pelleting the crypts/villi/organoids by centrifugation (1,000 x g for 5 min) before adding fresh wash solution.

### 4. Blocking Step

1. Make the blocking solution: add 10% secondary antibody species serum in PBS (optional: add 0.1% detergent).
2. Pellet the crypts/villi/organoids by centrifugation (1,000 x g for 5 min) and remove the supernatant.
3. Add 5 - 10 mL of the blocking solution depending on number of samples required (see note below). At this stage, split the crypts/villi/organoids solution into individual tubes (1.5 mL low binding microcentrifuge tubes with each tube containing 0.2 - 1 mL) for staining with the different antibodies. NOTE: Each isolated crypts/villi fraction will give between 5 - 10 samples that can be used for different labeling combinations depending on the size of the pellet. Ideally, a pellet size between 2 - 4 mm is required for each sample. Different fractions can be combined at this stage as crypts and villi can easily be identified (Figure 2).
4. Incubate in blocking solution at RT on a tube rotator for 1 h.

### 5. Primary Antibody Incubation

1. Dilute the primary antibodies (see **Table of Materials**) in PBS containing 10% serum and 0.1% detergent. Between 100 to 200 µL primary antibody solution is required per microcentrifuge tube sample.
2. Remove the blocking solution by centrifugation (1,000 x g for 5 min).
3. Resuspend the crypts/villi/organoid pellet in the primary antibody solution and incubate at 4 °C overnight using a tube rotator (20 RPM) to keep the crypts/villi/organoids in suspension.
4. Next day: Bring the samples back to RT for 1 h on tube rotator (20 rpm).
5. Pellet the sample by centrifugation (1,000 x g for 5 min) and remove the primary antibody solution.
6. Add 1 mL of washing solution and resuspend the crypt/villus/organoid pellets.
7. Immediately remove the solution by centrifugation (1,000 x g for 5 min).
8. Add 1 mL fresh wash solution and spin the cells on a tube rotator (20 rpm) for 2 hours. Change the wash solution by centrifugation every 30 min as in step 5.7.

### 6. Secondary Antibody Incubation

1. Dilute the secondary antibodies (see **Table of Materials**) in PBS with 10% serum and 0.1% detergent. About 200 µL of secondary antibody solution is required per microcentrifuge tube sample. NOTE: Highly cross-absorbed antibodies should be used to reduce reactivity of secondary antibodies in mouse crypt/villus/organoid.
2. Centrifuge at 1,000 x g for 5 min to pellet the crypts/villi/organoids and resuspend the pellets in 200 µL of secondary antibody solution.
3. Spin the tubes on a tube rotator (20 rpm) at RT for 1 h.
4. Centrifuge at 1,000 x g for 5 min to pellet the crypts/villi/organoids and remove the supernatants (non-bound secondary antibodies).
5. Resuspend the pellet in wash solution and immediately centrifuge at 1,000 x g for 5 min to pellet the crypts/villi/organoids.
6. Remove the supernatant and resuspend the pellet in 1 mL of fresh wash solution.
7. Spin on a tube rotator (20 rpm) at RT for 1.5 - 2 h, changing the wash solution every 20 - 30 min as described previously in steps 6.3 - 6.6.

### 7. Nuclear Stain

1. Dilute the nuclear stain in wash solution (as per the manufacturer's protocol), such as DAPI or Hoechst. For example: DAPI stock solution (20 mg/mL) is diluted 1:10,000. The stock solution can be kept in aliquots at -20 °C.
2. Centrifuge at 1,000 x g for 5 min to pellet the crypts/villi/organoids and remove washing solution.
3. Resuspend the pellet in nuclear counterstain solution.
4. Spin on a tube rotator (20 rpm) at RT for 10 min.
5. Centrifuge at 1,000 x g for 5 min to pellet the crypts/villi/organoids, remove the nuclear stain solution, and resuspend the pellet in wash solution.
6. Spin on a tube rotator (20 rpm) at RT for 30 - 60 min, changing the wash solution every 10 min.

### 8. Mounting Isolated Crypts, Villi, and Organoids

1. Centrifuge at 1,000 x g for 5 min to pellet the crypts/villi/organoids and remove all the wash solution. NOTE: If the pellet disperses, then centrifuge again.
2. Add 2 drops of hard setting mounting media with antifade agent to the pellet.
3. Cut the end of a P200 micropipette and carefully resuspend the pellet in the mounting media. Avoid generating bubbles. NOTE: The isolated organoid cultures are very sticky; make sure to resuspend fully.
4. Use the micropipette to transfer the mixture of crypts/villi/organoids in the mounting media solution, and dispense in a line along the center of a microscope slide. NOTE: The line should not be longer than the cover-glass that is to be used. Check using a stereomicroscope whether the crypts/villi/organoids are well spread on slide and not clumped.
5. Carefully place a cover-glass over the top; avoid generating bubbles.
6. Place the glass slide in a slide book and store in a fridge overnight for the mounting media to set before analyzing on a confocal microscope. NOTE: For mouse antibody staining in mouse tissue, use the commercial immunofluorescence kit (see **Table of Materials**). Replace the blocking step (Section 4) with a 10 min block with protein blocking solution followed by a 1 h incubation with the blocking reagent that comes with the kit. Then at step 5.8 (in the middle of washing) add a 1 h incubation with fluorescence signal enhancer reagent. Then use the kit's reagent in fluorescent dilutant (other secondary antibodies can also be used in combination with this reagent). Incubate as above and proceed from step 6 with the rest of the protocol.

### 9. Fixation and Immuno-labeling of Organoids Within Basement Matrix

NOTE: Organoids destined for fixation and immuno-labeling while remaining within the basement matrix were generated in basement matrix domes on top of round glass coverslips in a 24-well plate (one dome per well). The organoid basement matrix domes were processed within the 24-well plate by the addition and removal of the various solutions.

1. Remove the medium from wells and add the fixative; leave for 1 h.
2. Remove the fixative and wash in PBS containing 1% serum and 0.1% detergent for 2 h, changing every 30 min.
3. Remove the wash solution and block in PBS with 10% serum and 0.1% detergent for 1 h.
4. Dilute the antibodies in PBS with 1% serum or PBS with 1% serum and 0.1% detergent.
5. Remove the blocking solution and add 100 - 200 μL primary antibody (see **Table of Materials**) solution to each well. NOTE: it is important to remove all the blocking solution so as not to dilute antibodies further.
6. Place plate in the fridge and incubate overnight at 4 °C
7. Then leave at RT for 1 - 2 h.
8. Remove the antibody solution and wash for 2 - 3 h changing the wash solution every 20 min.
9. Remove all wash solution and add 100 - 200 μL secondary antibodies diluted (see **Table of Materials**) in PBS with 1% serum; incubate for 1 h at RT.
10. Remove the secondary antibody solution and wash for 2 h, changing every 20 min.
11. Remove the wash solution and add DAPI solution; leave for 10 min at RT. Use DAPI stock solution (20 mg/mL) diluted at 1:10,000. The stock solution can be kept in aliquots at -20 °C.
12. Remove the DAPI solution and wash for 40 min, changing every 10 min.
13. Using forceps, remove the glass coverslip with the basement matrix dome and place on a slide with the basement matrix dome facing upwards; add a few drops of mounting media and then put a glass coverslip on top.
14. Place the glass-slide in a slide book and leave in fridge overnight for the mounting media to set.

## Representative Results

**Isolation of intestinal tissue for immuno-labeling** The described tissue isolation protocols for colon and small intestine were optimized for preservation and immuno-labeling of microtubules and associated proteins, but not for stem cell viability and organoid generation (Figure 1 and **Table 1**). The aim was to generate crypt and villus factions that were as clean (devoid of mucus and other tissue) as possible, while minimizing exposure to EDTA and cold to preserve structure and prevent depolymerization of microtubules with ice cold solutions, which induce depolymerization of all but stable microtubules. Figure 2 shows examples of images of Fractions 2 and 3 from isolated small intestinal tissue, with Fraction 2 containing a mixture of both villi and crypts (Figure 2A**, B**), while Fraction 3 contains mainly crypts (Figure 2C**, D**).

**Fixation and immuno-labeling of isolated intestinal tissue** The individual or combined fractions were then processed for fixation and immuno-labeled through a series of steps including fixation, detergent, blocking, antibody, and washing solutions, before re-suspending the final villi/crypts pellet in mounting media, transferring to slides, and covering with glass coverslips. The crypts and villi were then imaged on a confocal microscope.

Good preservation and labeling of microtubules and actin in both villi and crypts was achieved by the following: a combination of formaldehyde/methanol fixation at -20 °C, repeated washing in PBS containing 0.1% detergent and 1% serum and blocking in PBS with 0.1% detergent and 10% serum, followed by overnight incubation at 4 °C in primary antibodies and then 2 h in secondary antibodies at room temperature (Figure 3). Formaldehyde/methanol fixation also worked well for labeling +TIPs such as the EBs and CLIP-170 in isolated crypts and villi (Figure 4). EB3 accumulations at the plus-end of microtubules (known as comets) were evident in crypts (Figure 4A), while association along the lattice of stable microtubules could be seen in villi samples (Figure 4C). Distinct localization of CLIP-170 and p150^Glued ^(subunit of dynactin) was clearly evident at the apical n-MTOCs in isolated villi (Figure 4B). Fixation with the formaldehyde/methanol protocol did not consistently work for ninein localization in isolated intestinal tissue using our Pep3 antibody against mouse ninein. However, methanol fixation at -20 °C followed by the same washing and blocking solutions as for formaldehyde/methanol gave very good localization of ninein within isolated crypts and villi (Figure 5; reference^8^). Interestingly, while ninein is concentrated at the apical centrosomes some accumulation at the cell base was evident in some cells within isolated crypts (Figure 5). Whether this is due to non-specific labeling or a consequence of the isolation procedure delaying fixation (and thus affecting preservation) will need further investigation. However, methanol-fixed (-20 °C) cryostat sections of villi (see Figure 3bi in^8^) also revealed ninein at the cell base in some cells suggesting that ninein may also associate with a basal population of microtubules.

**Fixation and immuno-labeling of organoids isolated from basement matrix** Small intestinal organoids were generated and grown in basement matrix for three weeks or longer (Figure 6A; reference^6^,^15^). A cold (4 °C) cell recovery solution was used to isolate organoids from the basement matrix. The depolymerized basement matrix solution with organoids was transferred to tubes and centrifuged prior to fixation and immuno-labeling. This produced very clean preparations and allowed good access to the organoids for the various solutions. The differentiated cells within the organoid villus domains contain stable apico-basal microtubules; these labeled well in most cases (Figure 6B**, C**) and EB1 could also be seen along the microtubule lattice (Figure 6E; reference^13^). However, the cold cell recovery solution may result in depolymerization of the dynamic microtubules, which was evident in some samples by the lack of EB1 comets (which bind to the plus-end of growing microtubules) within the basal crypt domains (Figure 6F). In other samples, astral (dynamic) microtubules were preserved (Figure 6D). Organoid isolation prior to fixation and immuno-labeling also worked for junctional proteins, ninein, CLIP-170, and cell markers, such as mucin for goblet cells and chromogranin A for enteroendocrine cells.

Fixation and immuno-labeling of organoids within basement matrix Figure 7 shows an organoid at the cyst stage (**A-C**) and at an early stage of crypt development (**D**), both fixed in formaldehyde/methanol and immuno-labeled for microtubules and ninein. Good microtubule preservation and labeling as well as labeling for ninein at apical n-MTOCs were evident. Figure 8A, B shows a crypt domain within a day 6 organoid fixed with the methanol protocol and labeled for microtubules and EB1. Good preservation of microtubules and EB1 comets was evident suggesting preservation of dynamic microtubules.

Organoids were also fixed and immuno-labeled while remaining within the basement matrix. The disadvantages of this procedure may be poor penetration of fixative and trapping of antibodies within the basement matrix (Figure 8B), although in both cases less frequently when 0.1% detergent was included in the fixative and/or wash solutions. In addition, 4% PFA did not preserve the basement matrix well but caused it to dissolve, although this was less so with 1% PFA. Methanol fixation, on the other hand, sometimes induced organoid collapse.

Labeling with some antibodies such as against the stem cell marker Lgr5 and Paneth cell marker CD24 proved unsuccessful with the 4% PFA, methanol, or formaldehyde/methanol protocols. However, fixing the organoids within the basement matrix with 1% PFA in PBS with 0.1% detergent at room temperature did result in labeling for both Lgr5 and CD24 (Figure 9).

**Figure:**
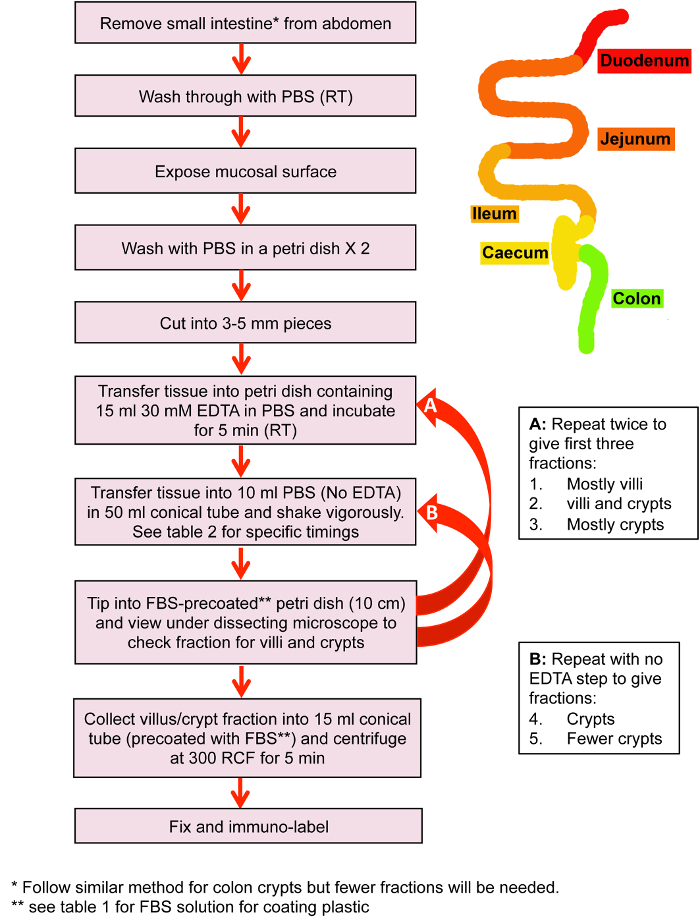

**Figure 1: Isolation of small intestinal villi and crypts.** Flow diagram of the key steps in small intestinal villus and crypt isolation prior to fixation and immuno-labeling. Please click here to view a larger version of this figure.

**Figure:**
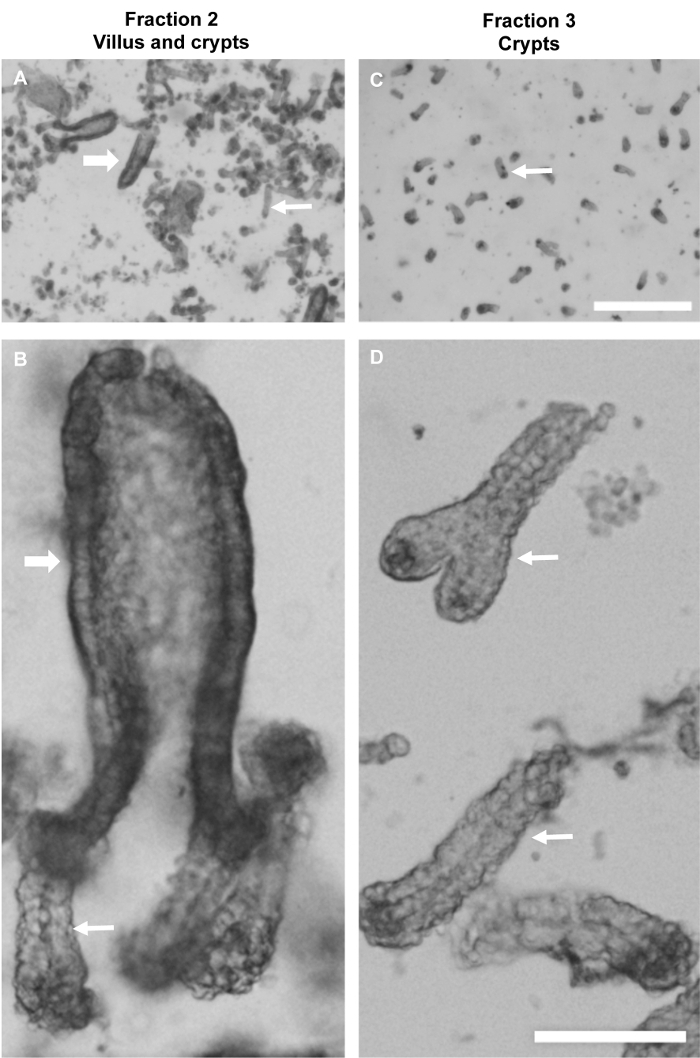

**Figure 2: Isolated villi and crypts from mouse small intestine.** Brightfield microscope images of intestinal fractions showing villi (large arrows) and crypts (small arrows). (**A, B**) Fraction 2 contains a mixture of villi and crypts, and preservation of the morphology on the villus and crypt is evident in **B**. (**C, D**) Fraction 3 shows isolation of crypts and absence of villi and intact crypts including a bifurcated crypt in **C**. Scale bars = 500 μm (A, C); 100 μm (B, D). Please click here to view a larger version of this figure.

**Figure:**
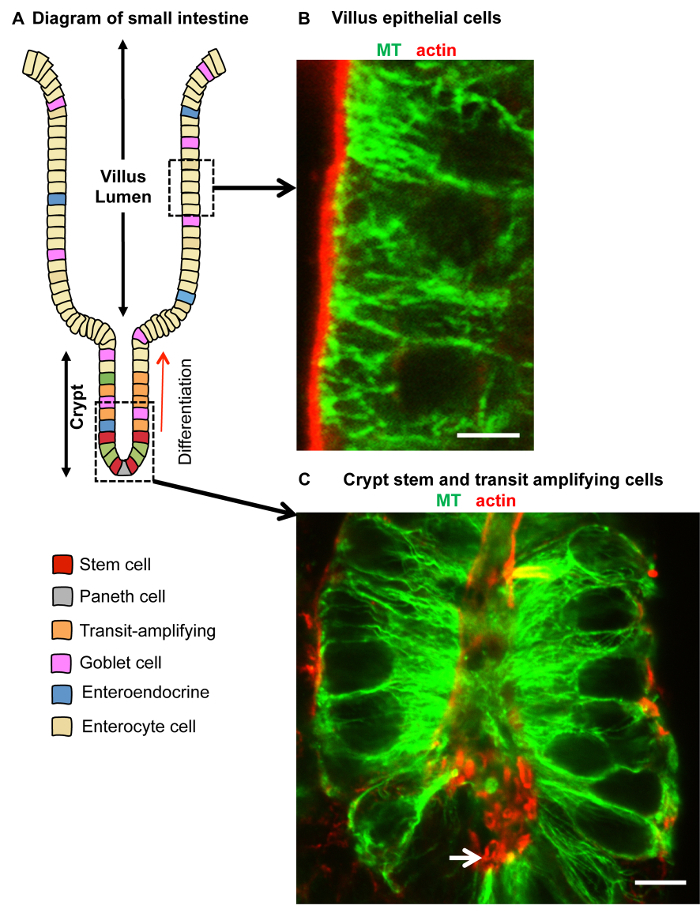

**Figure 3: Isolated small intestinal villus and crypt fixed in formaldehyde/methanol and immuno-labeled for microtubules and actin.** (**A**) Schematic of the villus and crypt epithelium with different cell types indicated. The highlighted boxes indicate the representative regions imaged in **B** and **C**. (**B, C**) Confocal optical sections through part of a villus (**B**) and basal crypt (**C**) isolated from the small intestine using 30 mM EDTA and fixed in formaldehyde/methanol, washed in PBS containing 1% goat serum and 0.1% detergent, blocked in PBS containing 10% goat serum and 0.1% detergent, and labeled for microtubules with rat monoclonal anti-tubulin antibody (green) and for actin with rabbit polyclonal anti-β-actin antibody (red). Well preserved apico-basal microtubule bundles are evident in both villus and crypt cells, and actin can be seen concentrated in the apical region facing the lumen (arrow). Scale bars = 5 μm. Please click here to view a larger version of this figure.

**Figure:**
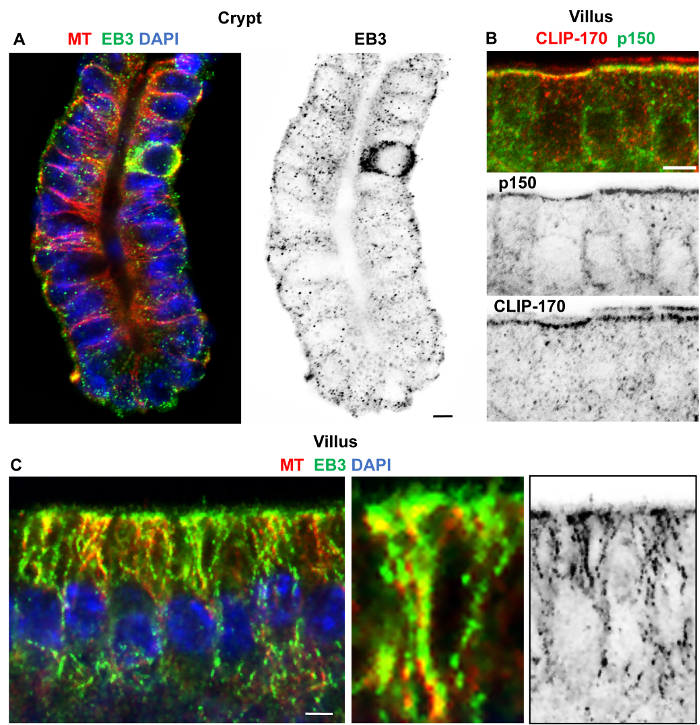

**Figure 4: Isolated small intestinal crypt and villi fixed in formaldehyde/methanol and immuno-labeled for microtubules, EB3, p150^Glued^, and CLIP-170.** Confocal optical sections of crypt and villi regions isolated from the small intestine using 30 mM EDTA and fixed in formaldehyde/methanol, washed in PBS containing 10% goat serum and 0.1% detergent, blocked in PBS containing 10% goat serum and 0.1% detergent, and immuno-labeled. (**A**) Crypt labeled with rabbit polyclonal α-tubulin antibody (red) and rat monoclonal EB3KT36 antibody (green) and stained for DNA with DAPI (blue) showing apico-basal microtubules and EB3 comets. The inverted single channel image clearly shows EB3 comets throughout the basal crypt cells suggesting good preservation of dynamic as well as stable microtubules. (**B**) Villus epithelial cells labeled with rabbit polyclonal CLIP-170 antibody (red, see also reference^16^) and mouse monoclonal p150^Glued^ antibody (green) showing apical co-localization. Inverted single channel images are shown below. (**C**) Villus cells labeled with rabbit polyclonal α-tubulin antibody (red) and rat monoclonal EB3-KT36 antibody (green) and stained for DNA with DAPI (blue) showing apico-basal microtubules with EB3 along the lattice. The EB3 lattice association is highlighted in the enlarged image while the inverted single channel image suggests both EB3 comets and lattice association. Scale bars = 5 μm. Please click here to view a larger version of this figure.

**Figure:**
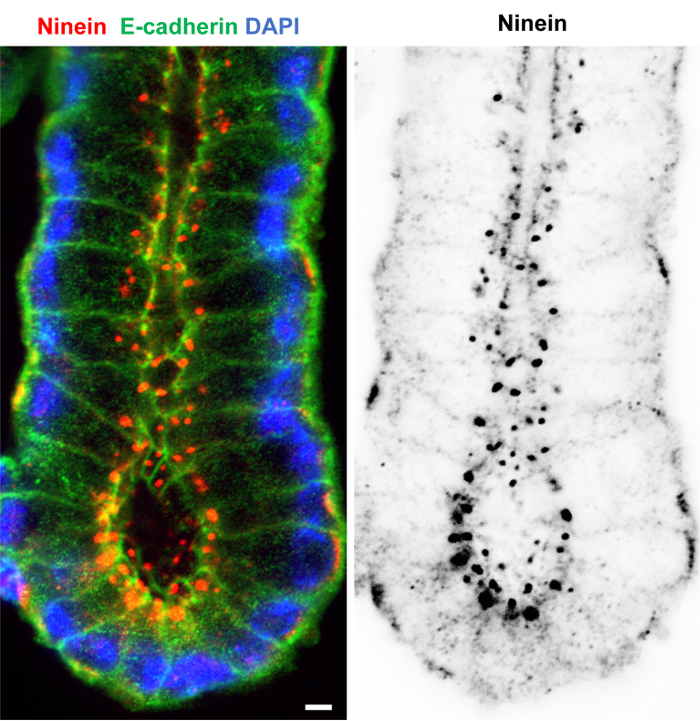

**Figure 5: Isolated colon crypt fixed in methanol, immuno-labeled for ninein and E-cadherin, and stained with DAPI.** Confocal optical section of the basal and transit-amplifying region of a crypt isolated from the colon using 3 mM EDTA and fixed in methanol, washed in PBS containing 1% goat serum and 0.1% detergent, and blocked in PBS containing 10% goat serum and 0.1% detergent. The crypt was labeled with rabbit polyclonal ninein antibodies (Pep3, see also reference^8^, red) and mouse monoclonal E-cadherin antibody (green) and stained with DAPI (blue). The inverted single channel image shows ninein only. The image shows a well-preserved crypt with E-cadherin revealing the outline of the individual cells and ninein concentrated at the apical centrosomes. It suggests good penetration of fixative and antibodies, and preservation of antigenicity. Scale bar = 5 μm. Please click here to view a larger version of this figure.

**Figure:**
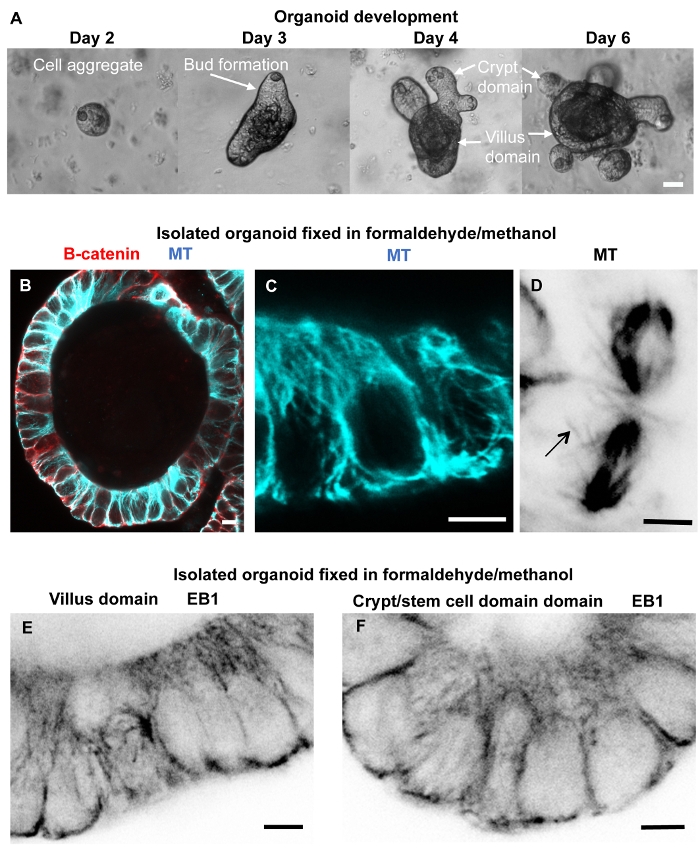

**Figure 6: Organoid development, fixation, and immuno-labeling of isolated organoids.** (**A**) Phase contrast images showing different stages of organoid development from cell aggregates to cyst with bud initiation and fully formed organoids with crypt and villus domains. (**B-F**) Confocal optical sections through organoids isolated from basement matrix using cell recovery solution at 4 °C (10 min) followed by formaldehyde/methanol fixation, washing in PBS containing 10% goat serum and 0.1% detergent, blocking in PBS containing 10% goat serum and 0.1% detergent, and immuno-labeling for microtubules, β-catenin, and EB1. (**B**) Organoid cyst labeled for microtubules (blue) and β-catenin (red) revealing good microtubule preservation and labeling in most cells. (**C**) Distinct apico-basal microtubules are evident in these enlarged epithelial cells from an organoid cyst. (**D**) Dividing cells labeled for microtubules showing spindles including astral (dynamic) microtubules (arrow). (**E, F**) Villus domain (**E**) and crypt domain (**F**) organoid regions showing some EB1 labeling along the lattice of stable microtubules, especially in the villus, while very few EB1 comets are seen even within the basal crypt suggesting that dynamic microtubules have not been preserved. Scale bars = 20 μm (A); 2 μm (D); 5 μm (B, C, E-F). Please click here to view a larger version of this figure.

**Figure:**
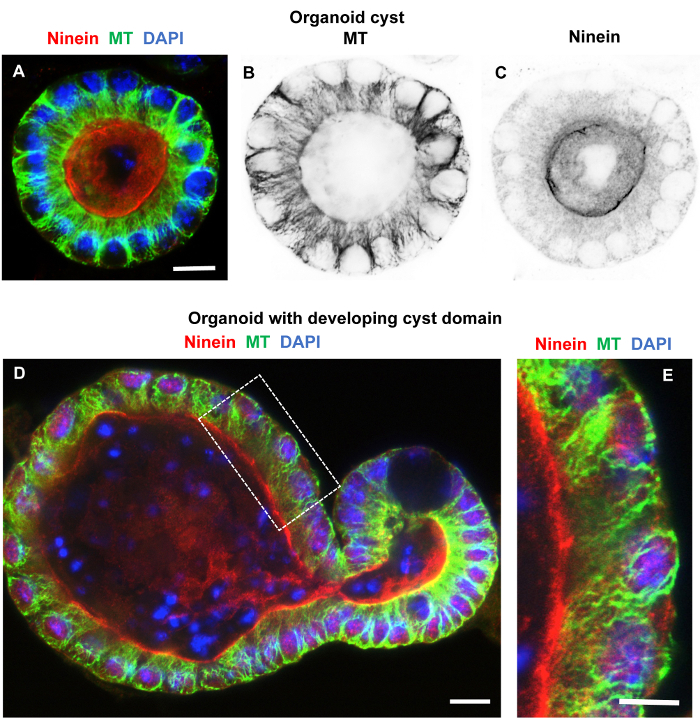

**Figure 7: Organoids fixed within the basement matrix in formaldehyde/methanol and immuno-labeled for microtubules and ninein.** Confocal optical sections of organoids fixed in formaldehyde/methanol, washed and blocked in PBS containing 10% goat serum and 0.1% detergent, and labeled while remaining in the basement matrix. (**A-C**) Organoid cyst labeled for microtubule (green) and ninein (Pep3; reference^8^, red) and stained with DAPI (blue) showing the merged image in **A** and inverted single channel images for microtubules (**B**) and ninein (**C**). The images show apico-basal microtubules and apical ninein localization, suggesting very good structural preservation of the organoid and penetration of antibodies as well as clearing of unbound antibodies. (**D, E**) Organoid with developing crypt fixed and labeled as above and again showing excellent structural preservation, labeling, and clearing of antibodies. Distinct apico-basal microtubules and apical n-MTOC ninein localization is evident and highlighted in the enlarged image (**E**) of the boxed region in **D**. Scale bars = 10 μm (A-D); 5 μm (E). Please click here to view a larger version of this figure.

**Figure:**
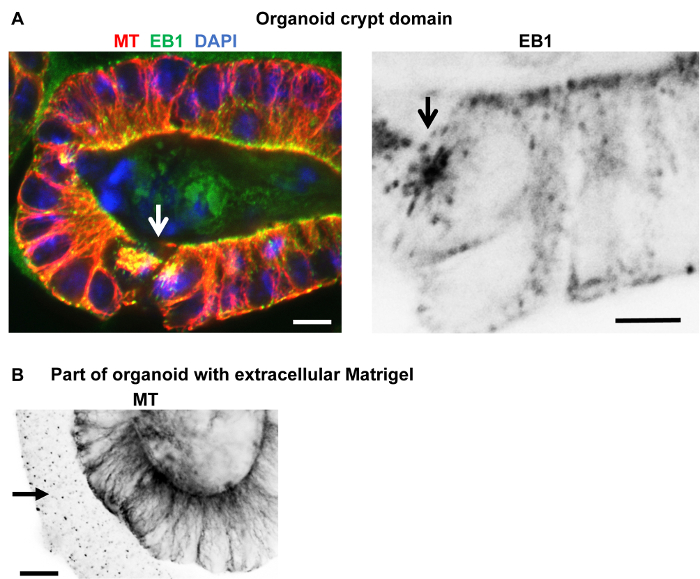

**Figure 8: Organoids fixed within the basement matrix in methanol and immuno-labeled for microtubules and EB1.** Confocal optical sections of organoids fixed in methanol, washed and blocked in PBS containing 10% goat serum and 0.1% detergent, and labeled, while remaining in the basement matrix. (**A**) Cyst domain from a fully developed organoid labeled with rabbit polyclonal α-tubulin (red) and mouse monoclonal EB1 (green) antibodies showing apico-basal microtubules, spindles (arrow) in two dividing cells and distinct EB1 comets. Some trapping of unbound EB1 antibodies is evident. However, good structural preservation and labeling of microtubules and EB1 is observed. The presence of EB1 comets suggests that dynamic microtubules have been preserved (**A**, invert). (**B**) Inverted image of organoid cyst region showing α-tubulin antibody labeling with considerable antibodies trapped within the surrounding basement matrix (arrow). Scale bars = 5 μm. Please click here to view a larger version of this figure.

**Figure:**
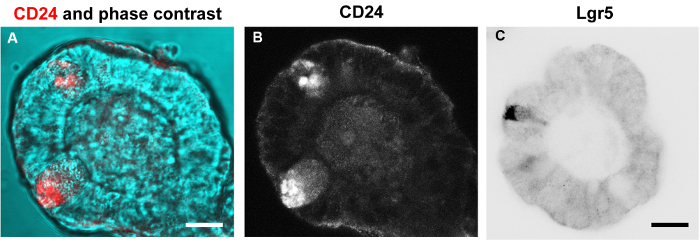

**Figure 9: Organoids fixed within the basement matrix in 1% PFA and immuno-labeled for Lgr5 and CD24.** Confocal optical sections of organoids fixed within the basement matrix in 1% PFA in PBS containing 0.1% detergent, washed in PBS with 1% goat serum and 0.1% detergent, and labeled with antibodies against Lgr5 and CD24. (**A, B**) Stem cell niche within a crypt domain showing Paneth cells positive for CD24 (red). The confocal and phase contrast images have been merged in **A**. **B** shows the single channel of CD24 labeling. (**C**) Stem cell region within a crypt domain showing a Lrg5 positive stem cell. Scale bars = 10 μm. Please click here to view a larger version of this figure.


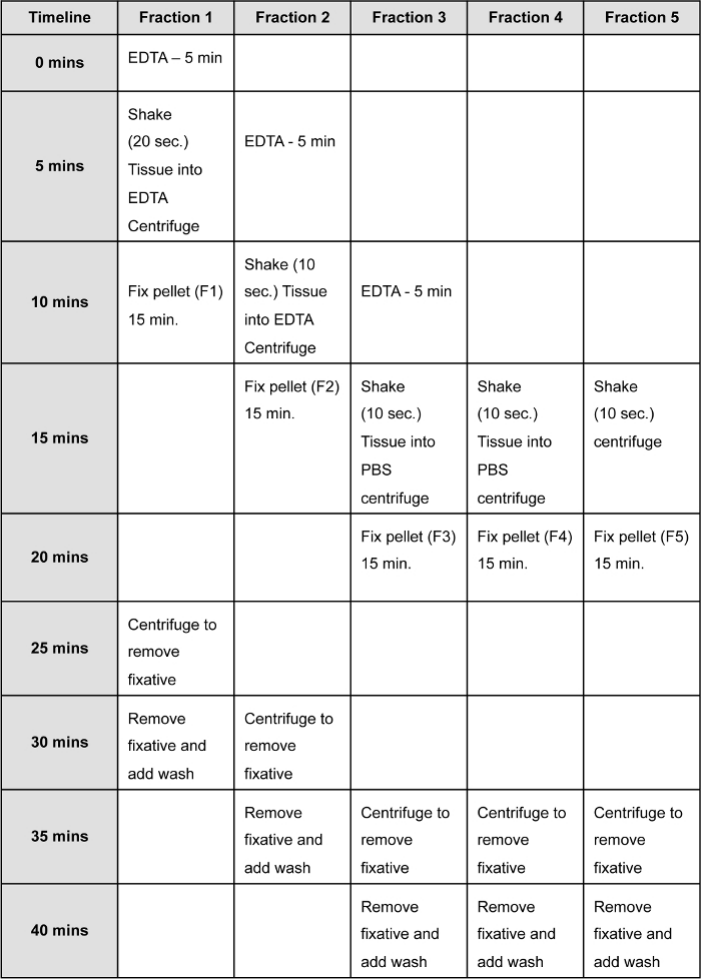
 **Table 1: Timeline of small intestinal crypt and villi isolation and fixation.** Please click here to view a larger version of this figure.

## Discussion

**Isolation of intestinal tissue** Isolation of small intestinal crypts and villi and colonic crypts involves exposing the mucosal surface, treatment with EDTA solution to loosen cell contacts, fractionation (shaking), and centrifugation. The presented intestinal villi/crypt isolation protocol has been modified from Belshaw *et al.* and Whitehead *et al.*^17^,^18^

**Exposing the mucosal surface** We have experimented with a number of approaches to expose the mucosal surface of the intestinal tract in the development of this procedure. A classic approach is to evert (turn inside out) the tube, usually in segments about 100 mm long, using a metal rod that is caught in a fold of the tissue at one end and then the remaining tube slid over the tube^19^. For mouse tissue, a metal rod (2.4 mm diameter) with rounded ends is ideal. This approach has the benefit of expanding the mucosal surface allowing better access to PBS and EDTA. We initially used this approach but moved to cutting the tube into short lengths (about 5 cm) and opening each section with dissecting scissors as this proved easier. This approach is appropriate if only a few intestines are required; but if more animals were to be used in an experiment then a purpose-built device for cutting open the tube longitudinally, as described by Yoneda *et al.*^14^ would be more efficient.

**Detachment of villi and crypts from the muscle layer** Initially we used 3 mM EDTA in PBS and relatively long incubation times of up to 60 min to loosen the mucosal surface from the underlying tissue^17^,^18^. At this concentration of EDTA we found an incubation time of 30 min was sufficient to loosen crypts from mouse colon. However, for the small intestine crypt/villus isolation we tried using more concentrated EDTA for a shorter time, which proved to be an efficient approach. All subsequent work was undertaken with tissue extracted using the 30 mM EDTA technique generating relevant fractions for villi or crypts. For crypts, we normally pooled fractions 3 - 5 before fixing but it is important to check whether these are the appropriate fractions as the timings will depend on a number of factors such as position along the intestinal tract, age of mouse, inflammation, previous diet, *etc.* Similarly, the length of time the tissue needs to be shaken following the EDTA treatment to be effective may vary under different conditions. The result is isolated tissue fractions containing a mixture of villi and crypts or mainly either villi or crypts (Figure 2). As there are no villi in the colon, the crypt extraction may be achievable in one step by shaking the tissue in the tube for 30 s. These fractions can then be fixed and processed for immuno-labeling.

**Isolation of intestinal organoids from basement matrix** Isolation of organoids from basement matrix domes can be achieved by using cell recovery solution. The solution works by depolymerizing the gelled basement matrix but the temperature needs to be 2 - 8 °C. A note of caution is that dynamic microtubules may not be preserved. Thus, for immuno-labeling of dynamic microtubules and +TIPs such as the EBs cell, recovery from basement matrix prior to fixation is not recommended. However, most of the microtubules in the differentiating organoid cells are relatively stable and these were preserved (Figure 6). It also worked well for immuno-labeling of centrosomal and junctional proteins as well as cell markers.

**Fixation protocols** Formaldehyde (freshly made from PFA) is a relatively rapid-acting fixative that forms reversible cross-links and 4% PFA works well for example, in immuno-labeling microtubules and gamma-tubulin and staining actin filaments with Phalloidin. More dilute PFA solutions such as 1% worked well for immuno-labeling for example, with the stem cell markers Lgr5 and Paneth cell marker CD24 within the crypt stem cell niche, while higher concentrations of PFA did not work.

The addition of glutaraldehyde gives better preservation of microtubules and the so called PHEMO fixation which consists of a mixture of 3.7% PFA, 0.05% glutaraldehyde and 0.5% detergent in PHEMO buffer (68 mM PIPES, 25 mM HEPES, 15 mM EGTA and 3 mM MgCl2)^2^ gives excellent preservation of microtubules without compromising antigenicity. It also works well for immuno-labeling gamma-tubulin, β-catenin, and E-cadherin, and staining actin filaments with phalloidin. However, in 3D tissue and organoid cultures, the PHEMO fixation produced inconsistent results and was therefore not used.

Methanol is a coagulant fixative that gives relatively good penetration and tends to preserve antigenicity. Fixation with 100% methanol (-20 °C) introduces some shrinkage, gives moderate morphology preservation, and works for microtubules, +TIPs, and many centrosomal antibodies including ninein in 2D cell cultures. However, some organoids collapsed when using this fixation method. In addition, penetration of antibodies through entire crypts, villi, or organoids was initially a problem but the addition of 0.1% detergent to the wash solution and prolonged washing achieved better results.

A combination of formaldehyde and methanol had previously been used by Rogers *et al.*^20^ to immuno-label EB1 in *Drosophila.* A fixation protocol based on a mixture of formaldehyde and methanol was therefore developed for intestinal tissue and organoids based on 3% formaldehyde and 97% methanol chilled to -20 °C, but omitting the 5 mM sodium carbonate from the mixture that was used by Rogers *et al*.^20^ In addition, samples were fixed in the freezer at -20 °C. This worked particularly well for immuno-labeling +TIPs, such as CLIP-170 and the EBs, but also proved excellent for fixing and immuno-labeling microtubules and actin within tissue and 3D organoids. Very good structural preservation was evident and antigenicity was preserved for several cytoskeletal and associated proteins as well as centrosomal proteins such as gamma-tubulin and ninein, although labeling for ninein worked more consistently with methanol fixation.

## Disclosures

The authors declare no competing financial interests.
